# Production of microencapsulated cinnamon extract using spray drying and evaluation of its effects on bread quality

**DOI:** 10.1038/s41598-025-30691-1

**Published:** 2025-11-28

**Authors:** Reyhaneh Nemati, Mehdi Gharekhani, Sepideh Tariverdi, Hamed Hamishehkar, Hamid Bakhshabadi

**Affiliations:** 1 Department of Food Science and Technology, Ta.C., Islamic Azad University, Tabriz, Iran; 2 Pharmaceutical Technology Laboratory, Drug Applied Research Center, Tabriz University of Medical Sciences, Tabriz, Iran; 3 Department of Agriculture, Minab Higher Education Center, University of Hormozgan, Bandar Abbas, Iran

**Keywords:** Bread enrichment, Cinnamon, Spray drying, Maltodextrin, Gum arabic, Biochemistry, Biotechnology, Chemistry, Materials science

## Abstract

Natural antioxidants can improve the oxidative and sensory properties of bread, but their instability limits their functionality. Encapsulation effectively addresses this issue. Therefore, this study aimed to evaluate the effect of spray drying on the physicochemical properties of cinnamon extracts encapsulated with different wall materials, including maltodextrin (MD), gum arabic (GA), and their combination. In addition, the effects of different levels (1, 2.5, and 5%) of the best microcapsule formulation, as well as the use of 1 and 10 mL concentrations of aqueous cinnamon extract (EX), were investigated on selected quality attributes of bread. Results indicated that MD samples exhibited higher process efficiency (39.20%), bulk density (0.49 g/cm^3^), solubility (96.98%), encapsulation efficiency (76.32%), total phenolic content (40.25 mg/100 g), and DPPH free radical scavenging ability (56.78%) compared to GA samples. Additionally, MD samples had lower moisture content (2.41%) and hygroscopicity (10.21%). Microscopic images of the spray-dried powders revealed larger particle size for GA samples. Based on the measured characteristics, the sample coated with MD was selected as the best microcapsule. The bread containing 5% microcapsule extract displayed the highest total phenol content (7.45 mg/100 g) and DPPH free radical scavenging ability (20.63%). Considering other results, particularly overall sensory acceptance, it can be inferred that enhancing the bread with up to 2.5% of microencapsulated cinnamon extract enhances its antioxidant properties without greatly impacting sensory quality.

## Introduction

There has been a significant increase in the popularity of functional foods over the past decade. This is because people’s nutrition preferences have changed tremendously, with a growing awareness of the specific health benefits of functional food ingredients^1,2^. Functional foods are foods that have potential beneficial effects on health beyond their primary nutritional value. They promote health and reduce the risk of disease. Functional foods have gained widespread popularity worldwide and are commonly referred to as “nutraceuticals” and “designer foods.” The concept of functional foods was initiated and regulated by the Japanese Ministry of Health and Welfare in the 1980s and then spread to North America and other markets. The amount of biologically active compounds in foods is very low, and therefore the consumption of fruits, animal products, and certain vegetables that are rich in bioactive compounds reduces the risk of various metabolic disorders and cancer^3^. Phytochemicals are plant bioactive compounds produced by plants to protect themselves. They can be extracted from various sources such as whole grains, fruits, vegetables, nuts, and medicinal plants. More than a thousand phytochemicals have been discovered to date. Some of the important phytochemicals include carotenoids, polyphenols, isoprenoids, phytosterols, saponins, dietary fibers, and polysaccharides. These phytochemicals have potent antioxidant activities and exhibit antimicrobial, antidiarrheal, anthelmintic, antiallergic, antispasmodic, and antiviral activities^4–6^. Additionally, certain phenolic compounds and flavonoids can inhibit key enzymes, such as α-glucosidase and α-amylase, which play essential roles in glucose absorption. Inhibiting these enzymes helps reduce postprandial blood glucose levels^7^. Cinnamon is one of the oldest and most widely known spices, used in cooking and traditional herbal medicine for thousands of years. In fact, it was among the first spices to reach the Mediterranean. Native to Sri Lanka and southern India, the cinnamon tree typically grows to a height of 7–10 m. Cinnamon is the name given to the bark of thin lateral branches harvested from the base of several tropical evergreen trees of the genus *Cinnamomum*^8^. Cinnamon is rich in antioxidants and can enhance the nutritional value of foods by improving their flavor, color, and quality. Additionally, cinnamon contains a significant concentration of bioactive compounds and phenolics, which have an exceptional ability to scavenge free radicals and anti-oxidation. Because of this, it can be used as a natural preservative. The antibacterial and antifungal properties of cinnamon also help to prevent harmful microorganisms^9^.

Bread is one of the most widely consumed cereal products, produced in various forms all over the world^10,11^. Enriching bread dough, which is prone to lipid oxidation and microbial growth, with natural antioxidants represents a suitable strategy to produce bread loaves with a higher degree of oxidative stability, leading to better sensory quality of bread during storage^12^. It has been reported that many food additives are used in the production of cereal products to prolong shelf life and improve their quality^13^. The structure and texture of bread depend on the gluten network of its dough, which is formed by proteins present in wheat flour. To provide the desired volume and texture of bread, a strong gluten network with elasticity and adequate gas retention capacity is required. However, the addition of phenolic substances can disrupt the gluten network and change the properties of the dough. Amino acids of gluten proteins can bind to phenols and change their structure and function^14^. These interactions can affect bread quality by impacting the elasticity, tensile strength, and gas-holding capacity of dough^15^. In addition to affecting dough properties, polyphenols can also modify the flavor, texture, and color of bread, all of which can have a significant impact on consumer acceptance^16^. On the other hand, the antioxidant properties of phenolic compounds can extend the shelf life of bread by preventing lipid oxidation during storage. Plant phenols offer numerous benefits, but their incorporation into the bread baking process requires careful optimization to balance their effects on physicochemical and sensory properties. While low concentrations may enhance the antioxidant capacity and quality of bread, high levels of plant polyphenols can damage the gluten network and disrupt the proper texture and specific volume of bread^17^.

However, bioactive materials face several challenges in practical applications. Low water solubility (fat-soluble nutrients) or sensitivity to environmental factors (UV, pH, heat, and gastrointestinal tract) can lead to reduced stability or even loss of biological activity ultimately affecting their absorption, utilization, and bioavailability in the human body. To overcome these limitations, encapsulation technology has emerged as an effective solution^18^. In recent years, microencapsulation technologies have increased in importance in the food industry, especially in the development of functional foods with high added-value ingredients^19^. Encapsulation is the technology of entrapping solid, liquid, or gaseous materials in capsules that release their contents at controlled rates and under specific conditions. This process includes the steps of forming a wall around the bioactive compound, preventing leakage, and avoiding the encapsulation of undesirable compounds in the capsule^20^. In the field of food processing, microencapsulation technology plays a major role in preventing food spoilage, loss of volatiles, and loss of essential oil properties. Simultaneously, this technology offers a practical solution for stabilizing and improving the management of these bioactive materials for the enrichment of new food products such as dairy products, beverages, and bakery products^19^. Various techniques have been used to encapsulate polyphenolic compounds, such as spray drying, spray cooling/chilling, extrusion, fluidized bed coating, coacervation, liposome entrapment, inclusion complex formation, centrifugal suspension separation, lyophilization, crystallization, and emulsion, among others^20,21^. The advantages of spray drying include the ability to work with heat-sensitive materials, fast drying speed, high throughput and efficiency, good retention quality of microcapsules, and the possibility of using a wide range of formulations and wall materials^22^.

Furthermore, although maltodextrin-based encapsulation systems have been previously applied in food matrices for example, Meral et al. (2025) encapsulated nano-sized oils in maltodextrin as a fat substitute to improve the oxidative and physical properties of cookies, our work differs substantially in both scope and mechanism. Unlike their focus on lipid encapsulation and fat replacement, we encapsulated a phenolic-rich aqueous cinnamon extract and evaluated not only encapsulation efficiency and particle morphology but also the post-baking stability of phenolics, antioxidant capacity, and bread texture, demonstrating the ability of maltodextrin to protect hydrophilic bioactives during baking^23^. On the other hand, while various researchers have explored the incorporation of plant extracts into bread^24,25^, our study introduces an innovation by utilizing cinnamon extract and, more importantly, by comparing three different wall materials: maltodextrin (MD), gum arabic (GA), and a combination of both (MD-GA), an approach that has not been thoroughly investigated in previous studies. We hypothesized that the choice of wall material would impact the physicochemical properties of the microencapsulated extract and, consequently, the sensory and functional quality of the bread. Therefore, this study focused on the microencapsulation of 10% aqueous cinnamon extract through spray drying using various wall materials and assessed the effects of the optimal formulation on key bread quality characteristics.

## **Materials and methods**

### Materials

In this study, cinnamon sticks were obtained from the local market in Tabriz, Iran. Wheat flour (Sanboran, Iran), active dry yeast (Molas Iran Company, Iran), flour improver (S500, Golnan Puratos, Iran), maltodextrin with a DE of 18–20 (Letra, Iran), gum arabic, ethanol with 96% purity, gallic acid, methanol, Folin-Ciocalteu reagent, DPPH, and NaCl solution were obtained from Merck, Germany.

### Preparation of aqueous extract from cinnamon

Initially, impurities and waste materials were removed from cinnamon by hand. The cinnamon was then crushed using a hammer mill (model TS-1500 – Toos Sheken Khorasan, Iran) and passed through a sieve with a mesh size of 40 (0.4 mm). Subsequently, 90 g of cinnamon powder was mixed with distilled water at 80 °C in a 1:10 ratio and gently stirred at the same temperature for 40 min. The resulting mixture was filtered through paper filters (Whatman No. 1) and then subjected to centrifugation (Heraeus Holding GmbH, Hanau, Germany) at 4000 g^26^.

### Preparation of microcapsules by spray drying

To prepare the mixture, the active ingredients were combined with an appropriate amount of wall materials (MD, GA, and MD + GA in a 1:1 ratio) to achieve a ratio of 10 parts wall material to 1 part core material. It is important to note that these compounds were mixed in distilled water at an initial ratio of 10% w/v. To ensure complete dissolution and hydration, all samples were refrigerated for 6 h prior spray drying. The spray drying process was conducted using a co-flow regime in a laboratory-scale spray dryer (Dorsatech, Tehran, Iran) with an inlet air temperature set at 140 °C. A peristaltic pump (operating at a pressure of approximately 1 bar) connected to a fluid atomizer with a 0.7 mm diameter orifice maintained a constant flow of liquid feed to the dryer. The drying chamber had an internal diameter of 480 mm and a height of 1000 mm. The encapsulated powders (EPs) were collected using a powder recovery system. These operating conditions were selected based on the results of several preliminary tests^25,27^.

### Tests performed on microcapsules

#### Moisture content

The moisture content of the samples was determined using the AOAC method (No. 943.06). Two grams of the sample were weighed into containers that had previously reached a constant weight in an electric oven (Memmert, Germany). The sample was then dried in an oven at a temperature of 100 ± 3 ° C for 3–5 h. After cooling in a desiccator, the moisture content was calculated using Eq. 1.

1 $$\:\mathrm{M}\mathrm{o}\mathrm{i}\mathrm{s}\mathrm{t}\mathrm{u}\mathrm{r}\mathrm{e}\:\mathrm{c}\mathrm{o}\mathrm{n}\mathrm{t}\mathrm{e}\mathrm{n}\mathrm{t}=\frac{({\mathrm{W}}_{1}-{W}_{2})}{m}\times\:100$$

In Eq. 1, W_1_ represents the initial weight of the empty container with the sample before drying, W_2_ is the weight of the container and sample after drying, and m represents the weight of the sample^28^.

#### Process yield

The Process yield (PY) was calculated by dividing the final powder weight obtained after each drying experiment (M_2_, based on dry weight) by the initial weight of the components used in preparing the mixture (excluding water) (M_1_, based on dry weight) according to Eq. 2^25^.

2 $${\rm PY}\:=\frac{{M}_{2}}{{M}_{1}}\times\:100$$

#### Bulk density

Bulk density was calculated by slowly adding 2 g of cinnamon extract powder to a graduated cylinder (10 mL) with markings at 0.1 mL intervals. The ratio of the powder’s mass to the volume it occupied in the graduated cylinder was then determined (in g/cm^3^)^29^.

#### Solubility

The solubility of the samples was determined following the method of Cano-Cauca et al. (2005) with slight modifications. A sample weighing 1 g (based on dry weight) was carefully added to 100 ml of distilled water and vigorously stirred at 700 rpm for 4 min using a magnetic stirrer (HS860, Iran). The resulting solutions were then transferred to tubes and centrifuged at 3000 g for 5 min. Subsequently, 25 ml of the supernatant solution was transferred to pre-weighed Petri dishes and promptly dried in an electric oven at 100 °C for 5 h. The solubility (%) was then calculated using Eq. 3^30^.

3 $$\:\mathrm{S}\mathrm{o}\mathrm{l}\mathrm{u}\mathrm{b}\mathrm{i}\mathrm{l}\mathrm{i}\mathrm{t}\mathrm{y}\:\left(\mathrm{\%}\right)\:=\frac{{S}_{2}}{{S}_{1}}\times\:100$$

In Eq. 3, S_2_ represents the weight of the dried supernatant after centrifugation, while S_1_ represents the weight of the dry matter of the original sample in g.

#### Hygroscopicity

Hygroscopicity was evaluated using the method described by Tsatsop et al. (2025). Samples weighing 1 g were placed in a container at 25 °C with a saturated NaCl solution (75.29% relative humidity). After one week, the samples were weighed, and hygroscopicity was expressed as grams of adsorbed moisture per 100 gram of dry matter (g/100 g of powder)^31^.

#### Particle size and morphology

The particle size and morphology of the samples were analyzed using scanning electron microscopy (SEM) according to the method outlined by de Barros Fernandes et al., 2014. The powders were affixed to a double-sided adhesive tape that was then attached to a scanning electron microscope stub (P-230, Pomentron, Seoul, Korea) measuring 1 cm in diameter and 1 cm in height. The stub was coated with gold using a vacuum evaporation apparatus, and the samples were examined under an electron microscope. The average particle size of the powder (based on 300 particles) was measured and reported in nanometers (nm)^32^.

#### Encapsulation efficiency (EE)

In order to measure the encapsulation efficiency, the surface of the powders was initially washed with ethanol to eliminate surface phenol from the capsules. Subsequently, the value of this index was calculated based on the total phenolic compounds (TPC) content of the powders, as detailed in “Total phenolic content (TPC)”, relative to the quantity of phenolic compounds present in the feed solution (prior to spray drying) using Eq. 4^25^.

4 $$\:\:\:\:\:\:\:\:\:\:\:\:\:\:\:\:EE\left(\%\right)=\frac{TPC\:in\:powders}{TPC\:in\:feed}\times\:100$$

#### Total phenolic content (TPC)

To determine the total phenolic content (TPC), a calibration curve of gallic acid in methanol was first obtained using the method presented by Capanzi et al. (2000). In this study, 50% methanol was used to dissolve the powders (with a final total solid content of 10%). The sample and solvent were then transferred to a 15 mL centrifuge tube, vortexed for 1 min, and sonicated for 20 min in an ultrasound bath (Agilent, USA) at 50 Hz, 75% intensity, and 150 W power at room temperature. This process was repeated, and the dissolved extracts were subsequently centrifuged at 2500 rpm for 10 min. The resulting supernatant was used for the measurement of phenolic compounds. In this method, a constant concentration of extracts was transferred to test tubes. Subsequently, 2.5 ml of Folin-Ciocalteu’s reagent diluted 10-fold with distilled water was added to the tubes. Then, 2 ml of sodium carbonate solution (7.5 g/100 ml) was added to the mixture and distilled water was added to reach the desired volume. After keeping the tubes at room temperature for 30 min, the absorbance of the resulting blue color was read by a spectrophotometer (Biochrom, UK) at a wavelength of 765 nm. To prepare a standard calibration curve, different concentrations of gallic acid (ranging from 10 to 400 mg/100 g) were used, from which Eq. 5 was derived^33–35^.

5 $$\:TPC=\frac{A-0.031}{0.005}$$

In Eq. 5, TPC (mg/100 g) is calculated from the absorbance (A) measured with a spectrophotometer, where 0.005 and 0.031 are the slope and intercept of the gallic acid standard curve, respectively.

#### DPPH free radical scavenging ability

After removing surface phenol using the 2-3-8 method, one gram of EPs was dissolved in 10 mL of distilled water and thoroughly stirred. Next, 3.9 mL of ethanolic DPPH solution (15 mM) was added to 0.1 mL of this solution. The samples were then centrifuged for 5 min at 4000 rpm. After incubating the samples in the dark for 40 min, their absorbance was measured at a wavelength of 517 nm. Finally, the free radical scavenging ability was calculated using Eq. 6.

6 $$\:\mathrm{D}\mathrm{P}\mathrm{P}\mathrm{H}\left(\%\right)=\frac{AC-AS}{AC}\times\:100$$

In Eq. 6, AS represents the absorbance of the sample, while AC represents the absorbance of the control without the sample (negative control, ethanolic DPPH sample)^36,37^.

### Bread preparation

In this study, a standardized baking program was used to ensure that all samples were prepared as medium crust bread. Control bread (without extract and encapsulated cinnamon extract powder) was made with wheat flour (100 g), water (55.5 mL), flour improver (S500, 1 g), active dry yeast (2 g), and salt (1.5 g) using an automatic bread maker (Iran). The total baking time was 2 h and 10 min, which included 15 min of kneading, 30 min of initial fermentation, 10 min of intermediate fermentation after punching down the dough (85 g for each mold), 50 min of final fermentation, and 25 min of baking at 220 °C. After baking, the loaves were cooled and stored in plastic bags until further analysis. The control sample was produced without any added encapsulated powder. Samples MD1, MD2.5, and MD5 contained 1%, 2.5%, and 5% of the best-prepared microcapsules, respectively, which were used as flour substitutes. Additionally, samples EX1 and EX10 were prepared with 1 and 10 mL of aqueous cinnamon extract, respectively, for further investigation^25,38^.

### Bread characteristics

#### Moisture content

Once the bread samples had cooled to room temperature, 2 g of the bread crumbs were taken and their moisture content was measured using the method described for EP samples (“Moisture content”) in this study. This test was conducted on the first and third days after baking.

#### Bread crust hardness

The evaluation of bread texture using the TPA method was conducted on the day of baking and on the third day of production. Initially, 2 × 2 × 2 cm bread crumb samples were prepared. These samples were then analyzed for texture using a Brookfield Texture Analyzer (model LFRA-4500, USA). Bread hardness was determined by measuring the maximum force required to penetrate a cylindrical probe with a 25 mm diameter to a compression level of 70% of the initial height, at a speed of 60 mm/min^10^.

#### Total phenolic content (TPC)

The total phenolic content (TPC) of bread samples was determined using the method described by Tan et al. (2015). To prepare the samples for polyphenol extraction, the bread was first dried at 40 °C for 24 h and then ground into powder with a particle size of 0.4 mm using a grinder. Subsequently, one gram of the ground bread samples was mixed with 10 mL of distilled water and magnetically stirred at 700 rpm for 1 h. After stirring, the samples were centrifuged at 4000 g for 15 min, and the supernatant was collected for further analysis. The total phenolic content was calculated according to the method described in “Total phenolic content (TPC)” and the results expressed as mg/100 g basis^34^.

#### DPPH free radical scavenging ability

After preparing bread samples for the measurement of phenolic compounds as outlined in “Bulk density”, the DPPH free radical scavenging ability was tested using the method described in “DPPH free radical scavenging ability”.

#### Sensory evaluation

This evaluation was conducted by 13 semi-trained individuals who were familiar with sensory evaluation techniques. The baked samples were provided to the evaluators after 2 h. The sensory properties of the bread were assessed in terms of shape and size, porosity and hollowness, firmness and softness of the texture, chewiness, smell, taste, and flavor. Each property was assigned a ranking coefficient of 4, 3, 3, 2, 2, 4, and 4, respectively. The evaluation coefficient for each property ranged from very bad (1) to very good (5). With this information, the overall acceptability was calculated using Eq. 7^39^.

7 $$\:Q=\frac{\sum\:(P\times\:G)\:}{\sum\:P}$$

Where Q = overall acceptance (number of quality of production samples), P = attribute rating coefficient, and G = attribute evaluation coefficient.

###  Statistical analysis

The research was conducted in a simple random design with three repetitions and the results were analyzed using SAS (9.4, New York, United States). Comparison of the mean data was done using Duncan Multipurpose Test with 95% reliability and Excel 2016 software was used to draw charts.

##  Results and discussion

### Properties of cinnamon extract

The results showed that the cinnamon extract contained 64.5 mg/100 g of gallic acid equivalents as total phenolic content and exhibited a DPPH free radical scavenging activity of 74.85%. Shahid et al. (2018) reported that the total phenol and DPPH free radical scavenging activity for cinnamon extract was 355.01 mg/g of gallic acid equivalents and 90.18%, respectively. The significant difference between these values and the findings of the present article is likely attributed to the different variety of cinnamon that was studied^40^.

### Physicochemical characteristic of encapsulated powders

#### Effect of wall type on moisture content

The moisture content in microcapsules, considered an impurity, is crucial for their quality and performance. For instance, if the moisture content in flavor microcapsules is too high, the viscosity of the shell materials increases, impacting the storage and release of flavor in related products^41^. Table 1 shows that the sample coated with gum arabic had the highest moisture content, while the sample coated with maltodextrin had the lowest. Additionally, the addition of gum arabic to maltodextrin in the cinnamon extract coating resulted in increased moisture content in the samples. This increase can be attributed to the branched structure and hydrophilic groups of gum arabic, which enhance its ability to bind water molecules from the environment^42^. In the food industry, it is crucial that the moisture content of food powders does not exceed 4%. The low water content can help keep the microcapsules in a glassy state, limiting adverse effects like molecular mobility and oxidation, and preventing the growth of microorganisms^43,44^. Therefore, all microcapsules prepared in this study exhibited this desirable relative humidity, essential for the stability of the powders during storage and their application in the food industry. Consistent with the present results, Zahara et al. (2024) also demonstrated that the use of gum arabic increased the moisture content of microcapsules containing Cosmos caudatus Kunth compounds compared to maltodextrin. Furthermore, a combination of these two gums resulted in higher moisture content compared to samples using maltodextrin alone as a coating^45^.

#### Effect of wall type on process yield

The results (Table 1) showed that the type of wall material had a significant effect on process yield. The highest process yield was observed in samples coated with maltodextrin alone, while the lowest yield was in samples coated with gum arabic. There was no statistically significant difference between the sample coated with a combination of maltodextrin and gum arabic (*p* > 0.05). In the spray drying process, the selected wall material must ensure stability and durability of the encapsulated particles, substances, or compounds. It should also be cost-effective in terms of encapsulation efficiency and performance^46^. Maltodextrin, as a drying aid, increases the glass transition temperature of the mixture and forms a thin layer around the particles. This reduces particle adhesion and improves process yield compared to using gum arabic^47^. These findings align with previous research by Azkiyah et al. (2024), which found that process yield for Sardinella lemuru Smart Flavor increased with a higher ratio of maltodextrin to gum arabic. The researchers attributed this increase to the higher volume and dissolved solids^48^. Xin et al. (2022) also compared maltodextrin and liquid smoke in the microencapsulation of smoke powder food flavoring. They reported that maltodextrin led to a higher process yield^49^.

**Table 1:** Effect of wall type on some physicochemical properties of microcapsules containing cinnamon extract.

Indicators	Treatments		
	MD	MD-GA	GA
Moisture content (%)	2.41 ± 0.11^c^	3.00 ± 0.10^b^	3.55 ± 0.15^a^
Process yield (%)	39.20 ± 1.85^a^	35.95 ± 1.18^b^	34.13 ± 1.25^b^
Bulk density (g/cm^3^)	0.49 ± 0.01^a^	0.43 ± 0.02^b^	0.37 ± 0.01^c^
Solubility (%)	96.98 ± 0.23^a^	94.67 ± 0.31^b^	92.26 ± 0.16^c^
Hygroscopicity (%)	10.21 ± 0.20^C^	12.32 ± 0.43^b^	15.75 ± 0.52^a^

The data are the average of three replicates ± SD, and data with the same lowercase letters in each row indicate lack of significance at the 5% level.

#### Effect of wall type on bulk density

The bulk density of powders is influenced by various factors such as chemical composition, particle size, moisture content, processing, and storage conditions. Essentially, when the mass remains constant, density increases as volume decreases. Higher bulk density values indicate the need for smaller containers, making storage easier. Additionally, higher bulk density values suggest less air present, reducing vulnerability to oxidation^50^. Results from Table 1 show that samples using only maltodextrin in their wall structure had the highest bulk density, while those coated with gum arabic experienced a decrease in bulk density. In this study, microcapsules made with gum arabic had the lowest bulk density, likely due to the sticky nature of gum arabic, necessitating larger storage containers for gum arabic-coated samples^51^. Similar findings were reported by Sarabandi et al. (2019) when encapsulating eggplant extracts using maltodextrin, maltodextrin-gum arabic, and gum arabic as coating materials. These differences were attributed to the low viscosity of gum arabic, potentially leading to the formation of larger microcapsule particles^52^. The variations may also be linked to the chemical composition, molecular weight, and internal structural bonding of the coating materials^51^. Overall, the results highlight the significant impact of carrier type on density, consistent with findings by Zolqadri et al. (2025), who noted a substantial difference in density of red-garlic peptides when carrier materials were altered. They found that using maltodextrin increased sample density, likely due to higher soluble solid content in the samples^53^.

####  Effect of wall type on solubility

The evaluation of microcapsule solubility is essential to understand how microcapsules behave in different mediums, particularly in water. It helps determine whether the core material is released in the medium. Solubility is influenced by the type of wall material used for encapsulation, the production technique of the microcapsules, and the core concentration^54^. A comparison of means using Duncan’s range test (Table 1) revealed that the type of wall significantly impacted the solubility of the samples at a 5% level. The sample coated only with maltodextrin had the highest solubility, showing 4.87% more solubility than the sample coated only with gum arabic. Additionally, the combination of these two gums increased solubility compared to the sample with only gum arabic. Maltodextrin is widely used in the encapsulation industry due to its high solubility in water, often as an additive in drying processes^55^. Siti et al. (2023) demonstrated that the use of maltodextrin, with its abundance of hydroxyl groups, increased the solubility of microcapsules containing seaweed. This finding aligns with the results discussed in this section^56^. Zolqadri et al. (2025) also observed that microcapsules with a pure maltodextrin coating exhibited the highest solubility and fastest wettability. However, when maltodextrin was combined with other ingredients, the solubility of the samples decreased^53^.

#### Effect of wall type on hygroscopicity

Microcapsules have a tendency to absorb moisture from the environment when exposed to high relative humidity, a property known as hygroscopicity. This characteristic is crucial in determining the stability of the core material. The hygroscopicity of a microcapsule is largely influenced by the type of wall material used to encapsulate the core material, specifically its moisture absorption rate, which significantly impacts the behavior of the microcapsules during storage^54^. The study’s findings indicated that the sample coated with gum arabic exhibited the highest hygroscopicity, while the use of maltodextrin resulted in a decrease in this property (Table 1). Gum arabic demonstrates higher hygroscopicity as a wall material due to its numerous branches with hydrophilic groups that readily bind to water molecules. The chemical composition of wall materials composed of maltodextrin and gum arabic powders has been characterized by low hygroscopicity properties, with maltodextrin showing a high digestibility percentage^57^. Mohd Nawi et al. (2015) similarly found that the use of gum arabic increased the hygroscopicity of rhizocapsules containing anthocyanins from Ipomoea batatas, aligning with the results of the present study^58^. Conversely, Tomsone et al. (2020) demonstrated that microcapsules with higher molecular weight wall materials exhibited lower hygroscopicity compared to those with lower molecular weight wall materials, contrasting the findings of this study. This disparity suggests that the differences in hygroscopicity may be attributed to the type of microcapsule material and the varying operating conditions^59^.

#### Effect of wall type on encapsulation efficiency

Table 2 shows that the highest encapsulation efficiency was observed in the maltodextrin-coated sample, while the lowest was in the gum arabic-coated sample. The efficiency of microcapsule encapsulation is heavily influenced by the characteristics of the wall material, preparation methods, properties of the core material, and environmental conditions^60^. Both maltodextrin and gum arabic are commonly used as encapsulating agents in spray drying, each with unique properties that impact encapsulation efficiency^61^. Maltodextrin is known for its good solubility and ability to form a protective matrix, while gum arabic offers excellent emulsifying properties and film-forming ability^62,63^. Sources suggest that maltodextrin typically results in higher encapsulation efficiency due to its rapid formation of a matrix around the core material, minimizing the loss of volatile or sensitive compounds during the spray drying process^55^. Consistent with the findings in this section, Akdeniz and Şahin (2017) also demonstrated that using maltodextrin alone as a coating resulted in higher microencapsulation efficiency compared to gum arabic or a combination of the two materials^64^. Mutavski et al. (2025) similarly found that maltodextrin was more effective in microencapsulating black elderberry phenolic compounds than gum arabic or a combination of the two materials through spray drying, aligning with the results presented in this section^65^. In their 2018 study, Lee et al. showed that the use of maltodextrin increases the product transfer temperature, effectively reduces product losses, and prevents caking problems^66^. On the other hand, it has been found that maltodextrins are known to be exceptional heat protectants and play an important role in maintaining the integrity of anthocyanins during the encapsulation process^67^.

**Table 2:** The effect of wall type on encapsulation efficiency and antioxidant properties of produced microcapsules.

Indicators	Treatments		
	MD	MD-GA	GA
Encapsulation efficiency (%)	76.32 ± 0.32^a^	72.49 ± 0.33^b^	69.15 ± 0.87^c^
Total phenolic content (mg/100 g)	40.25 ± 0.43^a^	37.85 ± 0.19^b^	35.64 ± 0.21^c^
DPPH Free Radical Scavenging (%)	56.78 ± 0.56^a^	53.29 ± 0.73^b^	50.28 ± 0.29^c^

Data are the average of three replicates ± SD and different letters in each row indicate significant differences at the 5% level.

####  Effect of wall type on total phenolic content

The results showed that the total phenol content of the produced microcapsules varied from 35.64 to 40.25 mg/100 g, with the sample coated with maltodextrin having more total phenol than the other samples (Table 2). The decrease in the amount of phenolic compounds when more gum arabic was used in the coating can be attributed to the higher viscosity of gum arabic compared to maltodextrin. This resulted in an increase in the time for droplet formation and mixing of the core materials during the drying process, leading to decreased efficiency of microencapsulation and a lower content of phenolic compounds. Saberi et al. (2023) also found that when the ratio of maltodextrin was higher than that of tragacanth, the total phenol content of the microencapsulated samples was higher. They noted that the total phenolic content of all microcapsules was lower than the initial content in grape pomace before microencapsulation, indicating that even spray drying as a drying method leads to the destruction of a small portion of the phenolic compounds^68^. In contrast to our results, Medina-Jaramillo and López-Córdoba (2025) demonstrated that gum arabic, due to its molecular structure (a biopolymer composed of a highly branched sugar structure with a small proportion of covalently bound protein to the carbohydrate skeleton), has a greater ability to form protective layers and therefore more effectively preserve onion polyphenols than maltodextrin^69^. This difference was attributed to the type of microencapsulation method used (freeze-drying in their study) and the type of microencapsulated compounds. Robert et al. (2010), who microencapsulated pomegranate peel polyphenols using maltodextrin and soy protein isolate as wall materials, reported two possible mechanisms for the reduction and differences in phenolic compounds in powders produced with different carriers. First, depending on the type of capsule structure produced by the carrier and its microencapsulation efficiency, some polyphenols may be more susceptible to oxidative degradation, leading to degradation reactions and a more rapid loss of these polyphenols in the powder. Another hypothesis proposed for this phenomenon is that phenolic compounds present on the surface of the capsules are more susceptible to oxidation compared to polyphenols contained within the capsule structure^70^. It has also been reported that MD microcapsules can quench singlet oxygen and provide antioxidant protection for the parent material^62^.

#### Effect of wall type on DPPH free radical scavenging

As shown in Table 2, in line with the results for total phenols, the DPPH free radical scavenging ability of maltodextrin-coated samples was higher than that of other samples. Additionally, the use of gum arabic led to a decrease in the DPPH free radical scavenging ability of the samples. Sarabandi et al. (2019) also noted in line with these results that maltodextrin is more effective than gum arabic in preserving phenolic compounds during microencapsulation. Consequently, the DPPH free radical scavenging ability of eggplant peel extract coated with maltodextrin was higher than that of gum Arabic^52^. Akbarbaglu et al. (2025) demonstrated that using whey coating with maltodextrin is more effective in preserving phenolic compounds and has a natural ability to scavenge DPPH free radicals in date seed protein hydrolysates compared to other coatings, such as whey protein with gum arabic. When studying the effect of wall type (gum arabic and maltodextrin) on the ability to inhibit DPPH free radicals, various results were observed^24^. For instance, Akdeniz and Şahin (2017) found that wall type has an insignificant effect on the ability to inhibit DPPH free radicals in microencapsulated onion peel extract^64^. Conversely, Tolun et al. (2016) showed that using a combination of maltodextrin with gum arabic in a ratio of 8 to 2 is more effective in preserving phenolic compounds and consequently increasing the ability to inhibit DPPH free radicals in microencapsulated grape phenolic compounds. These differences may be attributed to variations in seed type as well as different processing conditions^71^.

#### Effect of wall type on microstructure and particle size of powders containing cinnamon extract

**Fig. 1:**
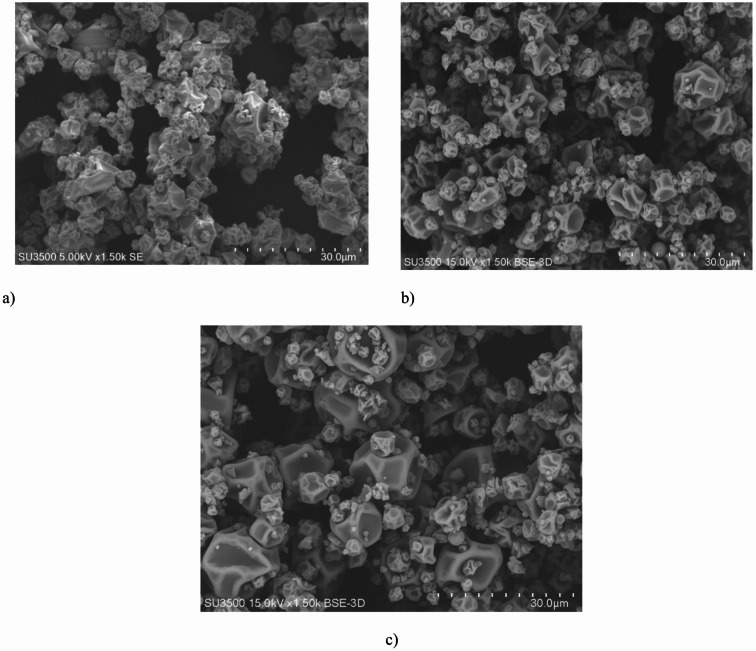
Effect of wall type (**a**) maltodextrin, (**b**) maltodextrin with gum arabic, and (**c**) gum arabic on the surface morphology of spray-dried cinnamon extract-containing particles.

**Fig. 2:**
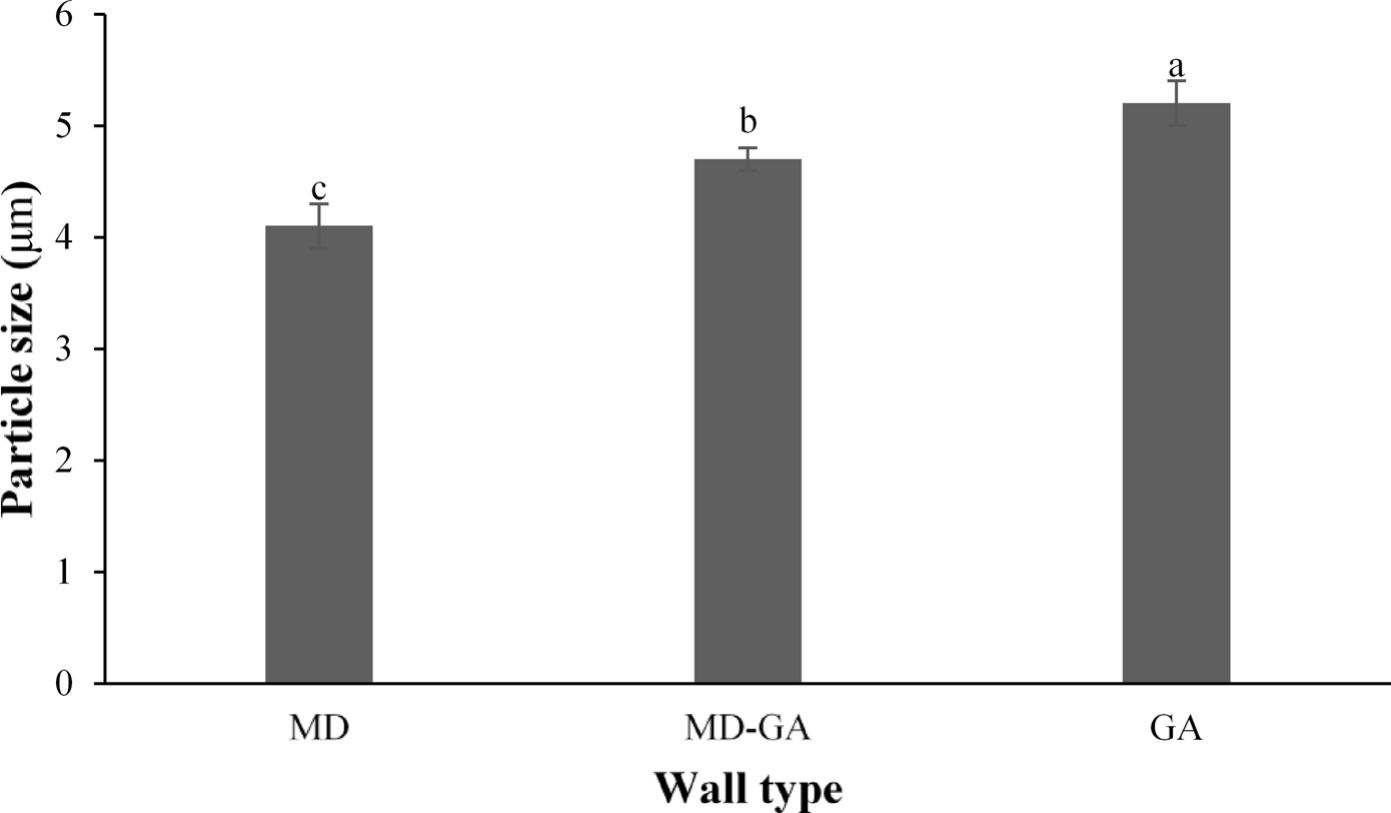
Effect of wall type (MD) maltodextrin, (MD/GA) maltodextrin and gum arabic, and (GA) gum arabic on the average particle size of spray-dried cinnamon extract-containing.

Figure 1 shows the particle structure of spray-dried cinnamon extract powders, displaying spherical, irregular, and wrinkled shapes. Electron microscope images revealed smaller particle sizes and less surface wrinkling in the particles produced with maltodextrin as a carrier compared to those produced with gum arabic. Various researchers have attributed the increase in particle size and greater wrinkling to the type, concentration, and viscosity of the carrier used. The production of particles with irregular and wrinkled surfaces is a common phenomenon in spray drying of various products, primarily due to the rapid formation of a skin on the droplets’ surface in the early stages of drying^72,73^. Similar to these studies, date powders produced with maltodextrin as a carrier exhibited relatively uniform, smooth, and spherical particles with numerous agglomerates. In contrast, samples obtained with gum arabic showed more particle shrinkage and less agglomeration compared to those prepared with maltodextrin. These findings highlight the varying effects of carriers and their combinations on the surface structure of the produced particles. Figure 2 illustrated that samples coated with maltodextrin had smaller particle sizes than microcapsules coated with gum arabic or a combination of maltodextrin and gum arabic. This difference can be attributed to the greater increase in viscosity in the feeds produced with gum arabic, resulting in coarser droplets and larger dried particles^74^. These results align with Deng et al. (2023) study, which reported that the size of maltodextrin-encapsulated purple corn anthocyanin microcapsules ranged from 8 to 80 μm^43^. These microcapsules were more uniform and significantly smaller than those encapsulated with a combination of maltodextrin and gum arabic (10–80 μm) or maltodextrin and whey protein isolate (10–100 μm). Microcapsules encapsulated with complex wall materials or a heterogeneous polydisperse system tend to aggregate and have larger sizes compared to microcapsules encapsulated with a single wall material or a homogeneous monodisperse system^75^.

### Physicochemical characteristics of bread samples

#### Moisture content

**Fig. 3:**
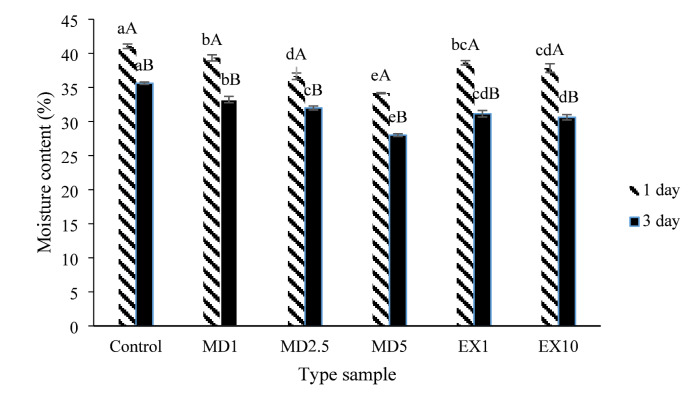
The effect of storage time and type of additive on the moisture content of the produced breads, similar lowercase letters for each storage day and similar uppercase letters for storage time for each formulation indicate non-significance at the 5 level.

Figure 3 illustrates the impact of varying ratios of free and microencapsulated cinnamon extract on the moisture content of the breads produced. The figure shows that as the percentage of microencapsulated cinnamon extract in the bread formulation increased, the moisture content of the breads decreased over both days of storage. The addition of free cinnamon extract also caused a decrease in moisture content compared to the control sample. However, adjusting the amount of free extract in the formulation did not significantly alter the moisture content of the samples (*p* > 0.05). Furthermore, it was observed that an increase in storage time correlated with a decrease in moisture content across all samples. The reduction in moisture content with a higher amount of microencapsulated extract in the bread formulation is attributed to hydrophilic polyphenol compounds competing with gluten protein for water absorption, ultimately leading to hardening. Additionally, other factors contributing to reduced water absorption include an increase in hydrophobic compounds in the extract, such as nonpolar amino acids and monoterpenes^76^. Pasrija et al. (2015) noted an increase in bread moisture content when using free green tea extract, attributing it to the presence of dietary fibers and polyphenols in the extract and their potential role in moisture retention^77^. However, the moisture content of breads produced with microencapsulated materials was lower than that of the control bread, as the wall and capsule components had a lower capacity to retain moisture within the gluten network of the bread. Additionally, during storage, moisture is lost from the bread surface, leading to drying and water migration from the core to the surface, resulting in decreased bread moisture and water activity^25^. Chen et al. (2021) also documented a decline in bread moisture content with prolonged storage time^78^.

#### Hardness of produced samples

**Fig. 4:**
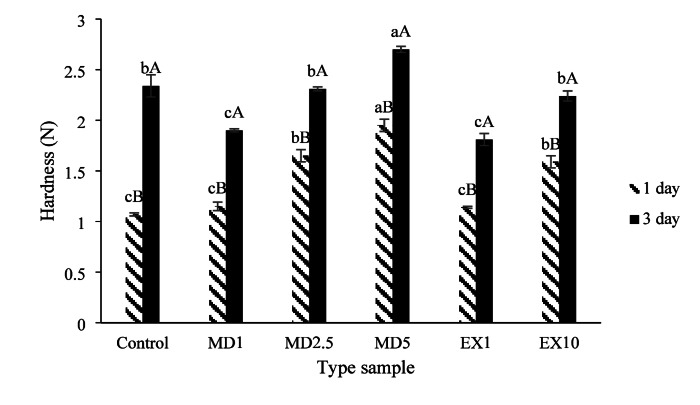
The effect of storage time and type of additive on the hardness of the produced breads, similar lowercase letters for each storage day and similar uppercase letters for storage time for each formulation indicate non-significance at the 5 level.

Figure 4 shows that on the first day of storage, the control sample and the samples containing 1% free and microencapsulated extract exhibited the lowest bread hardness. However, by the third day of storage, the lowest hardness was observed in the sample containing 1% free and microencapsulated extract. Additionally, increasing in extract concentration in both forms (microencapsulated and free), the hardness of the produced breads increased each day of storage (*p* < 0.05). It was also noted that as storage time increased, the hardness of the produced breads also increased. Akbarbaglu et al. (2024) suggested that increasing the proportion of microcapsules leads to increased bread hardness, attributed to the competition of microcapsules, starches, and proteins in the dough for water absorption^25^. This competition results in a decrease in the starch gelation process, preventing the formation of softer dough. In contrast, Ghasemi et al. (2022) found that the use of microcapsules is due to the high water absorption of the fiber molecules contained in them^79^. Therefore, the effect of materials such as microencapsulated bioactive compounds on firmness depends not only on their contribution but also on their properties (core material and matrix of microcapsules, particle size) as well as on the properties of the whole product, such as the presence or absence of gluten^80^. Textural changes during staleness and hardening of the bread crumb are closely related to starch reversion. Over time, moisture migrates from the interior of the bread to the crust, causing the texture to become firm, losing the original crispness and flavor of the bread^81,82^. Moisture is therefore linked to the firmness of the bread crumb. Initially, due to the high moisture content of the samples on day zero, the texture stiffness was low. However, over time, as soluble starch and moisture content decreased, crumb elasticity declined, leading to increased stiffness. Wang et al. (2025) noted that an increase in the level of extract containing phenolic compounds leads to increased stiffness due to non-covalent interactions between polyphenols and gluten proteins, resulting in the rearrangement of the secondary structure of gluten and a decrease in specific volume of bread^17^. On the other hand, sometimes a covalent bond may form between proteins and phenolic compounds, resulting in irreversible changes in protein properties and potentially leading to the formation of protein crosslinks^83^. which can increase the stiffness of the product. Lim et al. (2011) observed that the texture stiffness also increased with the addition of different percentages of turmeric powder. The decrease in stiffness on the third day of storage in samples containing 1% free and microencapsulated extract may be attributed to the maintenance of moisture content with increasing storage time^84^. Nouska et al. (2023) demonstrated that while adding various extracts increases hardness on the first day of storage, some compounds can reduce hardness over time due to their water-absorbing properties^85^.

####  Total phenol of produced samples

The results presented in Table 3 indicate that the sample with 5% microencapsulated cinnamon extract had the highest total phenol content (7.45 mg/100 g) among the samples produced. This was followed by the sample with 2.5% microencapsulated extract, which did not differ significantly compared to the sample with 10% free extract at the 5% level. The lowest total phenol content was found in the control sample and the sample with 1% free extract. The total phenol content of a product is largely influenced by the ingredients used. Using materials with a high polyphenol content results in higher levels of polyphenols in the final product. It is important to note that employing different techniques and materials, such as microencapsulation using various methods and matrix materials, can protect bioactive compounds from the negative effects of the external environment. This helps to reduce the loss of these compounds during thermal processing, thereby maintaining a high polyphenol content in the product. In contrast, the use of free compounds does not prevent losses^80^. A similar trend was observed in the enrichment of bread with microencapsulated Saskatoon berry extract, where the use of microencapsulation led to a significant increase in the polyphenol content of the product. Additionally, the study demonstrated that microencapsulation led to higher polyphenol levels compared to non-microencapsulated extract^86^. Costa et al. (2025) also found that increasing the pomegranate extract in bread formulations led to higher total phenol content in the samples, consistent with the findings of this study^87^.

####  DPPH free radical scavenging of produced samples

As shown in Table 3, the sample containing 5% microencapsulated extract exhibited the highest DPPH free radical scavenging ability (20.63%), whereas the control sample had the lowest level of this property. There was no statistically significant difference between the control sample and the sample containing 1% free cinnamon extract. Furthermore, the DPPH radical scavenging ability increased as the extract concentration in the formulation increased. Bagale et al. (2022) demonstrated that the antioxidant activity of bread is influenced by both technological factors and the type of additives used in the formulation. By incorporating curcumin nanoemulsion, they were able to enhance the ability to scavenge DPPH free radicals through encapsulation of curcumin and control of its solubility^88^. A study by Akbarbaglu et al. (2024) supported this correlation, showing that increasing the proportion of polyphenols, known for their high antioxidant activity, boosted the antioxidant capacity of the samples compared to both control samples and samples containing free extract^25^. The inclusion of substances with high antioxidant potential, such as polyphenols, generally leads to an increase in the overall antioxidant capacity of the product^89^. Similarly, maltodextrin-based nano-sized oil systems have been reported to significantly enhance oxidative stability and physical quality in baked products, functioning as efficient fat substitutes^23^. These findings align with those of Torgbo et al. (2022) in their study on bread made with rambutan peel extract, as well as with Pasrija et al. (2015), who found that microencapsulation of green tea extract preserved phenolic compounds in bread, thereby boosting its antioxidant properties^77,90^.

####  Overall acceptance of produced samples

In the case of bakery products, sensory characteristics are crucial for consumer acceptance, and texture, being particularly important for all types of bakery products. Texture includes several sensory attributes and is considered a key quality parameter for some products. Texture determines consumer perception and the value attributed to the products^91^. The results showed that the highest overall acceptance of the samples belonged to the control sample, which did not differ statistically from the samples containing 1% and 2.5% of microencapsulated cinnamon extract at the 5% level. However, with a further increase in the microencapsulated extract (more than 2.5%) in the formulation of the produced breads, the overall acceptance decreased. On the other hand, the lowest overall acceptance of the produced breads, due to its pungent and unpleasant taste, was related to the sample containing 10% of cinnamon extract. Shori et al. (2021) also stated, in line with the results of this section, that although the amount of phenolic compounds and antioxidant capacity of bread containing peppermint extract increased with increasing extract concentration, an increase of more than 2.5% of the extract resulted in a decrease in sensory evaluation scores^92^. Adding the extract in small amounts created a mild herbal aroma that was generally welcomed by the jury members. However, bread enriched with chamomile powder in larger amounts, although it had a richer taste, had a slightly bitter taste in the mouth that some jury members found less appealing. This bitterness is likely due to the higher concentration of bioactive compounds such as flavonoids in the powder, which contribute to a bitter taste. Additionally, adding medicinal plant powder leads to an increase in the firmness of the bread, which is also a reason for the decrease in overall acceptance scores^93^. Akbarbeglu et al. (2024) also showed that breads prepared with microencapsulated extracts received higher scores compared to breads prepared with free extracts^25^. The ratings were also close to the control sample level, indicating that the use of microencapsulated extracts did not affect consumer acceptance of the product in this case either. Bińkowska et al. (2024) also stated that the use of microencapsulation led to improved sensory properties of bread compared to the use of extracts^80^.

**Table 3:** Comparison of total phenol, DPPH free radical scavenging ability, and overall acceptance of breads containing different concentrations of cinnamon extract in both free and microencapsulated forms.

Indicators	Treatments					
	Control	MD1	MD2.5	MD5	EX1	EX10
Total phenol content (mg/100 g)	4.13 ± 0.14^d^	5.35 ± 0.19^c^	6.32 ± 0.11^b^	7.45 ± 0.10^a^	4.19 ± 0.16^d^	6.15 ± 0.26^b^
DPPH (%)	4.91 ± 0.27^d^	12.27 ± 0.56^c^	15.75 ± 0.43^b^	20.63 ± 0.89^a^	5.01 ± 0.33^d^	12.99 ± 0.67^c^
Overall acceptability	4.30 ± 0.20^a^	4.25 ± 0.17^a^	4.26 ± 0.19^a^	3.50 ± 0.25^b^	3.55 ± 0.15^b^	2.15 ± 0.10^c^

The data are the average of three replicates ± SD, and data with the same lowercase letters in each row indicate lack of significance at the 5% level.

## General conclusion

In this study, we evaluated the encapsulation of aqueous cinnamon extract in various wall materials (maltodextrin, gum arabic, and a combination of both) via spray drying. Our findings indicate that maltodextrin is the most effective wall material for spray drying cinnamon extract, as it provides better process efficiency, bulk density, solubility, total phenolic content, antioxidant activity, and particle morphology. Therefore, samples produced with maltodextrin were selected for bread fortification. Fortification of bread with different levels of encapsulated powder and aqueous cinnamon extract, compared to control bread, demonstrated that encapsulated cinnamon extract can effectively preserve phenolic compounds during processes such as baking. This enrichment of bread did not lead to a significant decrease in the overall acceptability of the bread samples produced. In conclusion, we recommend further research to investigate the cost of scaling up this process to an industrial level. The advantages of cinnamon extract microencapsulation and its potential application in the bakery industry make it a promising area for future exploration.

## Data Availability

The data that support the finding of this study are available from the corresponding author upon reasonable request.
